# Distinct In Vitro Differentiation Protocols Differentially Affect Cytotoxicity Induced by Heavy Metals in Human Neuroblastoma SH-SY5Y Cells

**DOI:** 10.1007/s12011-024-04342-x

**Published:** 2024-08-26

**Authors:** Jannatul Ferdous, Kiyotada Naitou, Mitsuya Shiraishi

**Affiliations:** 1 Department of Basic Veterinary Science, Joint Faculty of Veterinary Medicine, Kagoshima University, 1-21-24 Korimoto, Kagoshima, 890-0065 Japan; 2 Department of Pharmacology, Faculty of Veterinary Science, Bangladesh Agricultural University, Mymensingh, 2202 Bangladesh

**Keywords:** All-trans retinoic acid, Differentiation, Heavy metal, Insulin-like growth factor-I, SH-SY5Y cells, Toxicity

## Abstract

The SH-SY5Y cell line is widely used in neurotoxicity studies. However, the effects of inducing cell differentiation on the cytotoxic effects of heavy metals are unclear. Therefore, we investigated the effects of mercuric chloride (HgCl_2_), cadmium chloride (CdCl_2_), arsenic trioxide (As_2_O_3_), and methylmercury (MeHg) on SH-SY5Y cells differentiated in the presence of insulin-like growth factor-I (IGF-I) or all-trans retinoic acid (ATRA). Neurite outgrowth with distinct changes in neuronal marker expression, phenotype, and cell cycle was induced in SH-SY5Y cells by IGF-I treatment for 1 day or ATRA treatment for up to 7 days. The cytotoxic effects of HgCl_2_ decreased at lower concentrations and increased at higher concentrations in both IGF-I- and ATRA-differentiated cells compared with those in undifferentiated cells. Differentiation with IGF-I, but not with ATRA, increased the cytotoxic effects of CdCl_2_. Decreased cytotoxic effects of As_2_O_3_ and MeHg were observed at lower concentrations in IGF-I-differentiated cells, whereas increased cytotoxic effects of As_2_O_3_ and MeHg were observed at higher concentrations in ATRA-differentiated cells. Changes in the cytotoxic effects of heavy metals were observed even after 1 day of ATRA exposure in SH-SY5Y cells. Our results demonstrate that the differentiation of SH-SY5Y cells by IGF-I and ATRA induces different cellular characteristics, resulting in diverse changes in sensitivity to heavy metals, which depend not only on the differentiation agents and treatment time but also on the heavy metal species and concentration.

## Introduction

The term “heavy metal” refers to metallic elements characterized by relatively high densities, bioaccumulation potentials, and toxicity [1]. Rapid industrialization, expansion of agricultural arenas, domestic usage, and medication have increased the widespread distribution of heavy metals in the environment, seriously affecting human health and the environment [2]. After entering the human body through various roots, heavy metals cause life-threatening gastrointestinal, neurological, respiratory, and cardiovascular disorders [1, 3, 4]. The unique characteristics of the nervous system, such as the complex structure, composition of differentiated post-mitotic cells, and myelination, have made it susceptible to accumulating heavy metals readily to exert their toxicity [5]. According to the World Health Organization (WHO, 2020), mercury (Hg), cadmium (Cd), and arsenic (As) induce neurotoxicity and are major public health concerns [6, 7]. Inorganic and organic mercury are severe environmental pollutants with neurotoxicity, and methylmercury (MeHg), which has been identified as the cause of Minamata disease, is a well-established neurotoxicant [8–10]. Cadmium is considered to cause nervous system disorders, including Alzheimer’s disease, Huntington’s disease, and deterioration of cognitive functions [11]. Arsenic exposure has been reported to induce central nervous system impairment and peripheral neuropathy [12].

Human-derived SH-SY5Y cells are widely used to study neuroscience, including the neurotoxicity of heavy metals, Parkinson’s disease, Alzheimer’s disease, ischemia, and inflammation [13–16]. SH-SY5Y cells were subcloned from the SK-N-SH cell line established from human bone marrow metastatic neuroblastoma [17]. SH-SY5Y cells offer advantages over other neuronal cell lines, such as their human origin, high reproducibility, neuron-like characteristics, and differentiation capability [18]. SH-SY5Y cells exhibit catecholaminergic neuron characteristics, including tyrosine hydroxylase expression, dopamine-β-hydroxylase activities, and synthesis of both dopamine and noradrenaline [13, 19]. Furthermore, SH-SY5Y cells can differentiate into mature neuron-like cells upon treatment with differentiation-inducing agents [20]. Thus, these cells are a suitable model for neuroscience research on the proliferation and differentiation stages.

Although all-trans retinoic acid (ATRA) is the most common agent used to induce differentiation in SH-SY5Y cells, 12-O-tetradecanoyl-phorbol-13 acetate (TPA), B-27, AM580, metformin, and insulin-like growth factor-I (IGF-I) are also used to induce differentiation [21–24]. Differentiated SH-SY5Y cells display neurites with short cell bodies [20], cell cycle alteration [25, 26], and the expression of mature neuronal marker proteins, including synaptophysin (SYP), microtubule-associated protein 2 (MAP2), neuron-specific enolase, growth-associated protein 43 (GAP43), and β-tubulin III [22, 25, 27, 28]. The characteristics of cell lines, including SH-SY5Y cells, are affected by the cell source, culture media composition, and the protocol used to induce differentiation [13, 20, 29–31]. In addition, SH-SY5Y cells differentiate into different neuronal phenotypes, including dopaminergic or cholinergic phenotypes [20], depending on the agents used to differentiate the cells and culture conditions. Therefore, when studying neuronal function in vitro, it is necessary to select appropriate conditions and understand phenotypic characteristics to obtain the most accurate results compared with in vivo models.

Many studies on the cytotoxic effects of heavy metals have been conducted in SH-SY5Y cells [10, 13, 16, 32–34]; however, the effects of inducing cell differentiation and protocols on the sensitivity of SH-SY5Y cells to these heavy metals remain unclear. In this study, we selected ATRA and IGF-I as differentiation agents to explore the cytotoxicity of heavy metals because ATRA is the most common differentiation agent for SH-SY5Y cells, while IGF-I has been reported to induce neurite outgrowth of SH-SY5Y cells in a shorter period than ATRA and found to be neuroprotective against many toxic effects, such as MPTP/MPP + , oxygen and glucose deprivation, sodium arsenite, proteasome inhibitor epoxomicin, β-amyloid, and dopamine derivative salsolinol [35–39]. After evaluating the differentiated cell characteristics by morphological observation, expression of neuronal and phenotypic markers, and cell cycle analysis, the cytotoxic effects of mercuric chloride (HgCl_2_), cadmium chloride (CdCl_2_), arsenic trioxide (As_2_O_3_), and methylmercury (MeHg) were determined in undifferentiated and differentiated SH-SY5Y cells to determine the effects of cell differentiation protocols on heavy metal–induced cytotoxicity.

## Materials and Methods

### Reagents and Antibodies

Dulbecco’s modified Eagle’s medium/Ham’s F-12 (DMEM/F-12; 1:1), Dulbecco’s phosphate-buffered saline (D-PBS), SuperSep 10% gel, ATRA, and mercury (II) chloride (HgCl_2_) were purchased from Fujifilm Wako (Osaka, Japan). Human IGF-I was obtained from PeproTech (Cranbury, NJ, USA). Fetal bovine serum (FBS) was obtained from Mediatech (Woodland, CA, USA). Accutase, RIPA buffer, protease inhibitor cocktail, and Blocking One solution were purchased from Nacalai Tesque (Kyoto, Japan). FxCycle PI/RNase staining solution was purchased from Thermo Fisher Scientific (Carlsbad, CA, USA). The Cell Counting Kit-8 was obtained from Dojindo (Kumamoto, Japan). Methylmercury (II) chloride (MeHg) was purchased from Sigma (St. Louis, MO, USA), and cadmium chloride 2.5 hydrate (CdCl_2_) was purchased from Kanto Chemical (Tokyo, Japan). Arsenic (III) oxide (As_2_O_3_) was obtained from Alfa Aesar (Ward Hill, MA, USA). Immobilon Forte Western HRP substrate was obtained from Millipore (Burlington, MA, USA). The anti-MAP2 (17490–1-AP), anti-GAP43 (16971–1-AP), anti-β-tubulin III (10094–1-AP), anti-SYP (17785–1-AP), and horseradish peroxidase-conjugated anti-mouse IgG (SA0001-1), anti-rabbit IgG (SA00001-2), and anti-goat IgG (SA0001-4) antibodies were purchased from Proteintech (Rosemont, IL, USA). The anti-GAPDH (FL-335) antibody was purchased from Santa Cruz Biotechnology (Santa Cruz, CA, USA). Anti-TH (MAB318) and anti-ChAT (AB144P) antibodies were purchased from Millipore (CA, USA).

### Cell Culture and Differentiation

The human neuroblastoma cell line SH-SY5Y (ATCC, Manassas, VA, USA; passage 27) was cultured for up to five passages after acquisition from ATCC in DMEM/F-12 containing 10% heat-inactivated FBS (hiFBS) and antibiotics (100 U/mL penicillin and 100 µg/mL streptomycin). Cells were cultured in a humidified incubator at 37 °C and 5% CO_2_. Cells were plated at a density of 1.2 × 10^4^ cells/cm^2^ in 35-mm dishes 1 day before treatment with IGF-I or ATRA for cell morphology observation. Cells were plated 1 day before treatment with IGF-I or ATRA at a density of 6 × 10^4^ cells/cm^2^ in 35-mm dishes for western blotting and flow cytometry or in 96-well plates for cell viability assays. IGF-I or ATRA were dissolved in D-PBS containing 0.1% BSA or ethanol, respectively. After 8 h of serum starvation, the cells were incubated with DMEM/F-12 containing 50 ng/ml IGF-I and antibiotics for 1 day to obtain differentiated SH-SY5Y cells with IGF-I. In cell differentiation induction by ATRA, SH-SY5Y cells were incubated with DMEM/F-12 containing 10 µM ATRA, 1% hiFBS, and antibiotics for up to 7 days, and the media were changed every 3 days. Undifferentiated (vehicle-treated) and differentiated (IGF-I- or ATRA-treated) SH-SY5Y cells were exposed to different concentrations of heavy metals: 3–100 µM HgCl_2_, CdCl_2_, and As_2_O_3_ and 0.3–10 µM MeHg for 48 h. We conducted preliminary experiments in undifferentiated cells to determine optimum heavy metal concentrations, and selected concentration ranges across which concentration-dependent decreases in cell viability were observed.

### Cellular Morphology Observation and Neurite Length Measurements

SH-SY5Y cells were cultured in 35-mm dishes and differentiated by IGF-I or ATRA treatment as described above. Neuronal differentiation was confirmed by observing neurite outgrowth using an inverted microscope CKX53 (Olympus Optical, Tokyo, Japan) equipped with a digital camera (WRAYCAM-EL310; WRAYMER, Osaka, Japan) at × 10 or × 20 magnification. Images were captured using MicroStudio software (WRAYMER). Neurite lengths were measured using ImageJ software (NIH, Bethesda, MD, USA) using the “Freehand Line” and “Measure” tools. Neurites longer than twofold of the cell body’s diameter (> 50 µm) were considered for further calculation [21, 22]. Data were analyzed from three randomly selected fields per dish. At least 200 cells per field were considered for average neurite length measurement.

### Western Blot Analysis of Mature Neuronal Markers and Neurotransmitters

SH-SY5Y cells in 35-mm dishes were washed twice with ice-cold D-PBS and lysed with RIPA buffer containing protease inhibitors. The protein concentrations of lysate were quantified by the Bradford method. Cell lysates were boiled in a sample buffer and separated using SDS-PAGE (SuperSep 10% gel), followed by transfer to PVDF membranes. Membranes were blocked with Blocking One solution for 1 h at room temperature. After incubating PVDF membranes overnight at 4 °C with anti-MAP2 (dilution 1:20,000), anti-GAP43 (Dilution 1:20,000), anti-β-tubulin III (dilution 1:20,000), anti-SYP (dilution 1:25,000), anti-TH (dilution 1:3,000), anti-ChAT (dilution 1:3,000), or anti-GAPDH (dilution 1:5,000) antibodies, the membranes were incubated with secondary anti-mouse IgG (dilution 1:10,000), anti-rabbit IgG (dilution 1:10,000), or anti-goat IgG (dilution 1:10,000) antibodies for 1 h at room temperature. Protein bands were visualized using the Immobilon Forte Western HRP substrate on a LumiCube imager (Liponics, Tokyo, Japan). Densitometric analysis was performed using the ImageJ software. The expression level of GAPDH was used to normalize the expression of the other proteins. The expression of each neuronal marker and neurotransmitter in the control cells (no treatment) was considered 100%.

### Cell Cycle Analysis with Flow Cytometry

SH-SY5Y cells in 35-mm dishes were washed twice with D-PBS and harvested with 200 µL of accutase. Accutase was quenched by adding 1 mL of D-PBS, and the cells were collected in a tube and centrifuged to obtain cell pellets. After fixing the cells with ice-cold 70% ethanol for 30 min at 4 °C, the cells were rewashed with ice-cold PBS. Then, cells were stained with 500 µL of FxCycle PI/RNAse solution for 30 min in the dark at room temperature according to the manufacturer’s instructions. A BD FACSVerse (BD Biosciences, San Jose, CA, USA) flow cytometer was used for cell cycle analysis.

### Cell Viability Assay

SH-SY5Y cells were seeded into 96-well plates and treated with HgCl_2_, CdCl_2_, As_2_O_3_, or MeHg for 48 h. The cytotoxic effects of heavy metals were determined by a cell viability assay using a Cell Counting Kit-8, following the manufacturer’s instructions. Changes in the absorbance of WST-8 were measured at 450 nm using a MultiSkan microplate reader (Thermo Fisher Scientific, CA, USA). The cells not treated with heavy metals were considered 100% viable.

### Statistical Analysis

The results are expressed as mean ± SEM. Statistical analyses were performed by Student’s *t*-test for differences between means or by two-way analysis of variance (ANOVA) followed by post hoc analysis with Bonferroni or Dunnett’s test for multiple comparisons using R software (R-Project for Statistical Computing, www.r-project.org/). Statistical significance was set at *p* < 0.05, which was considered significant.

## Results

### Morphology of SH-SY5Y Cells Differentiated with IGF-I or ATRA

**Fig. 1:**
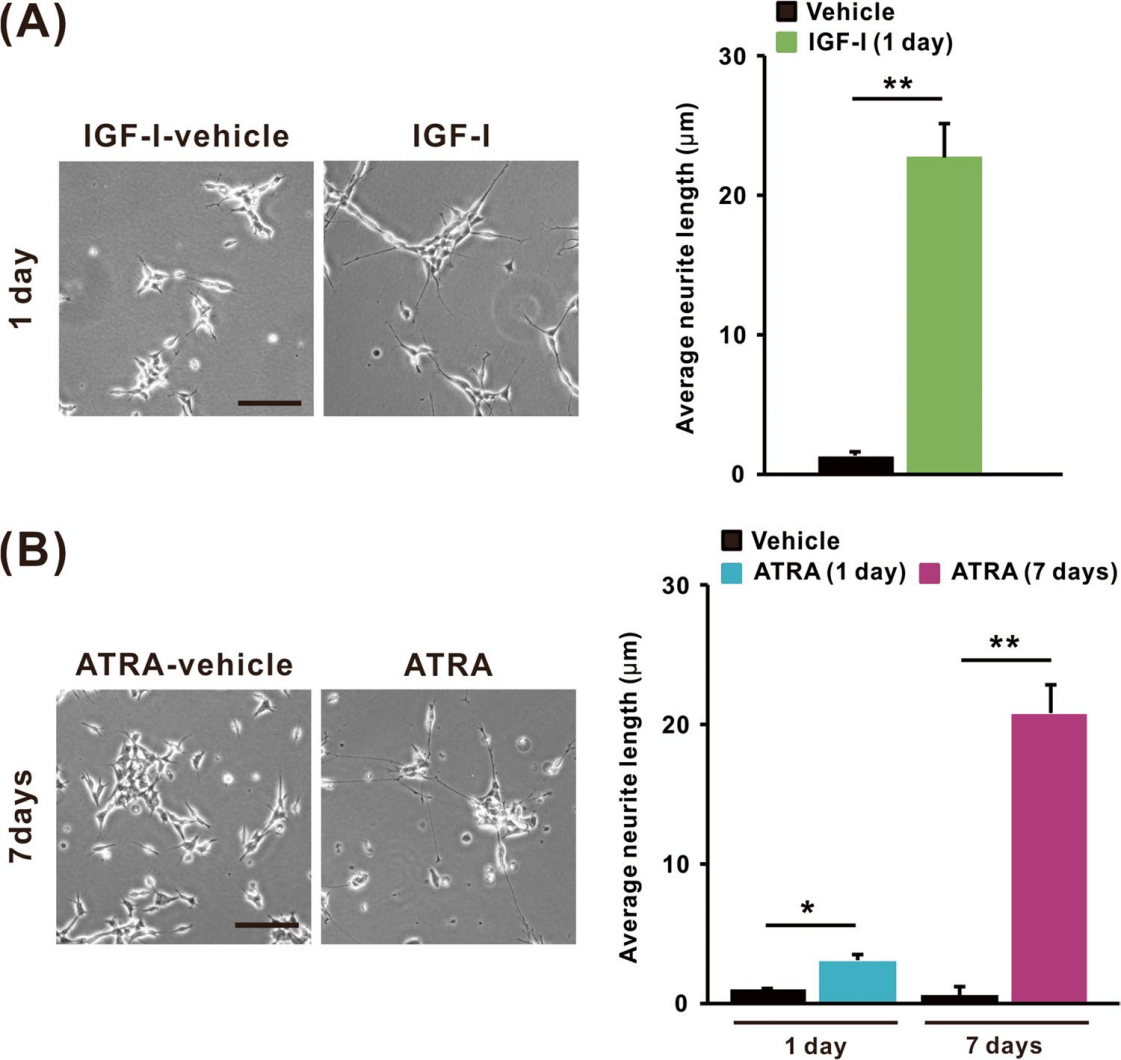
Morphology observation of SH-SY5Y cells differentiated with IGF-I or ATRA. Representative images of cell morphology and average neurite length of cells treated with **A** IGF-I vehicle and IGF-I (50 ng/mL) for 1 day and **B** ATRA vehicle and ATRA (10 µM) for 1 and 7 days. Scale bar is 100 µM. IGF-I was treated in DMEM/F-12 without hiFBS for 1 day, whereas ATRA was treated in DMEM/F-12 containing 1% hiFBS for up to 7 days. The result was expressed as mean ± SEM (*n* = 3). *p < 0.05, **p < 0.01 compared with vehicle-treated cells

IGF-I and ATRA treatments can differentiate SH-SY5Y neuroblastoma cells into neuron-like morphologies [21, 40]. Neurite outgrowth is a characteristic of differentiated SH-SY5Y cells. Morphological changes were observed in cells treated with IGF-I for 1 day or with ATRA for up to 7 days (Fig. 1). Exposure of SH-SY5Y cells to IGF-I (50 ng/mL) for 1 day induced neurite outgrowth (Fig. 1A), while obvious neurite outgrowth was induced by ATRA (10 µM) at 7 days after treatment (Fig. 1B). The average neurite length of cells treated with IGF-I for 1 day (22.7 ± 2.3 µm) was similar to that of cells treated with ATRA for 7 days (21.2 ± 2.0 µm), in which treatment with ATRA for 1 day caused only a slight increase in the average neurite length (Fig. 1A and B).

### Neuronal and Phenotypic Marker Proteins in SH-SY5Y Cells Differentiated with IGF-I or ATRA

**Fig. 2:**
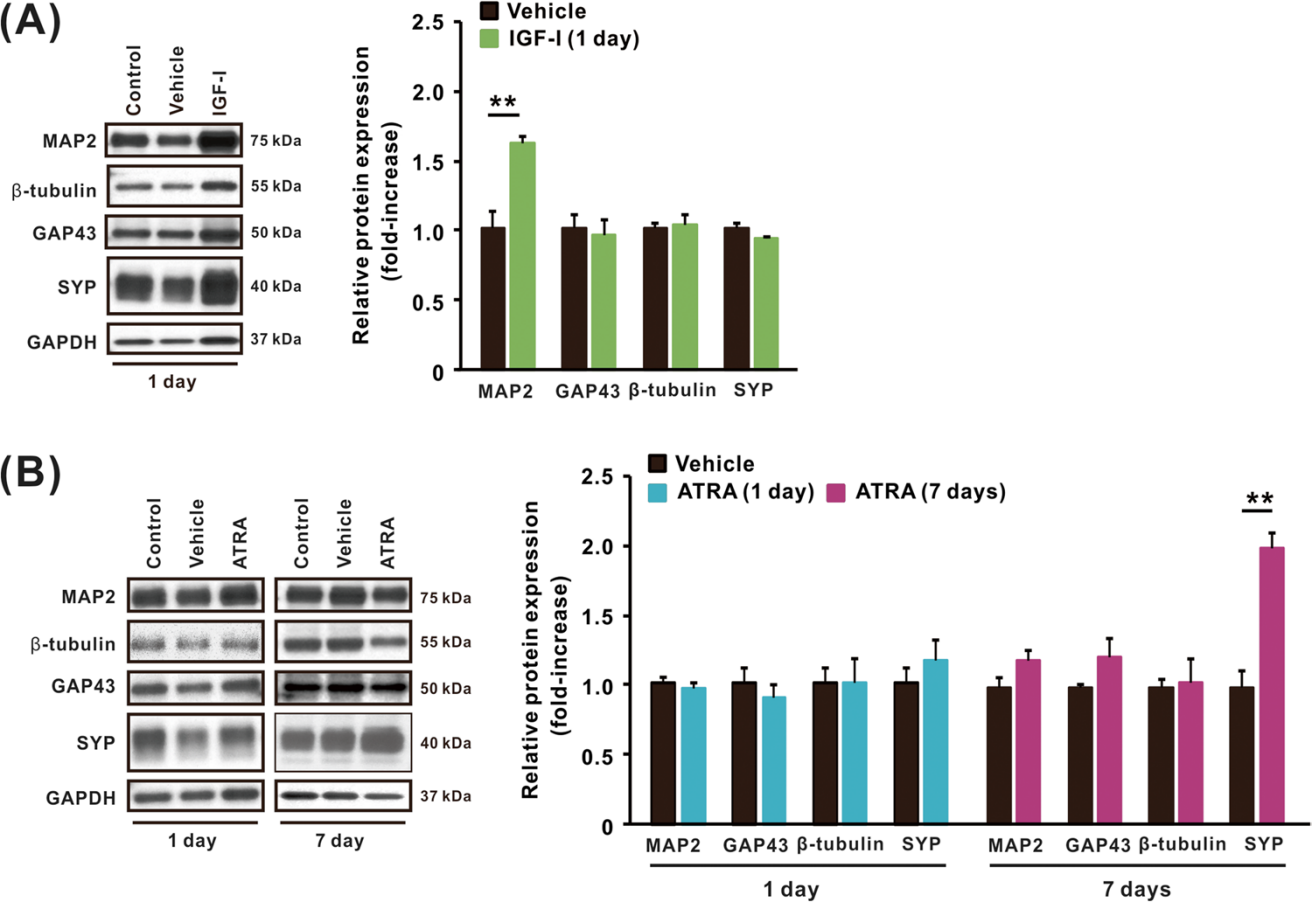
Expression of neuronal marker proteins in SH-SY5Y cells differentiated with IGF-I or ATRA. Representative western blot images and relative expression of neuronal markers including MAP2, β-tubulin, GAP43, SYP, and GAPDH in cells treated with **A** IGF-I vehicle and IGF-I (50 ng/mL) for 1 day and **B** ATRA vehicle and ATRA (10 µM) for 1 and 7 days. IGF-I was treated in DMEM/F-12 without hiFBS for 1 day, whereas ATRA was treated in DMEM/F-12 containing 1% hiFBS for up to 7 days. Relative expression levels of each protein were determined using densitometric analysis. Results are shown as mean ± SEM (*n* = 3). **p < 0.01 compared with vehicle-treated cells

We investigated the expression of neuronal marker proteins in undifferentiated and differentiated SH-SY5Y cells (Fig. 2). To compare the effects of IGF-I and ATRA on SH-SY5Y cell differentiation, the expression of mature neuronal marker proteins, including MAP2, β-tubulin, GAP43, and SYP, was measured using western blotting (Fig. 2A and B). MAP2 levels increased significantly in cells treated with IGF-I for 1 day compared with those in undifferentiated cells (Fig. 2A). Neuronal marker protein expression did not change after 1 day of ATRA treatment, but SYP increased significantly in cells treated with ATRA for 7 days (Fig. 2B).

**Fig. 3:**
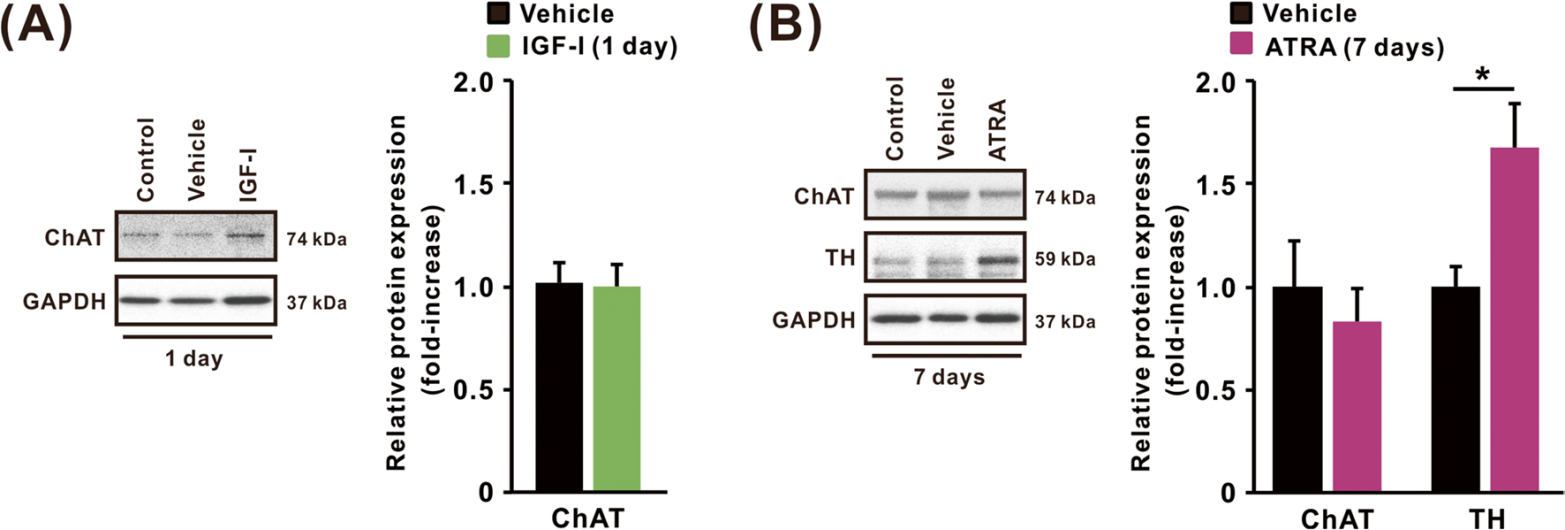
Expression of phenotypic marker proteins in SH-SY5Y cells differentiated with IGF-I or ATRA. Representative western blot images and relative expression of neuronal markers including ChAT and TH in cells treated with **A** IGF-I vehicle and IGF-I (50 ng/mL) for 1 day and **B** ATRA vehicle and ATRA (10 µM) for 1 and 7 days. Relative expression levels of each protein were determined using densitometric analysis. IGF-I was treated in DMEM/F-12 without hiFBS for 1 day, whereas ATRA was treated in DMEM/F-12 containing 1% hiFBS for up to 7 days. Results are shown as mean ± SEM (*n* = 3). *p < 0.05 compared with vehicle-treated cells

Since SH-SY5Y cells can be guided to dopaminergic or cholinergic neuronal phenotypes through different differentiation protocols [41], we extended our study to categorize differentiated cells into neuronal phenotypes. To identify the neuronal phenotypes of cells treated with IGF-I or ATRA, the expression of TH, a dopaminergic neuronal marker, and ChAT, a cholinergic neuronal marker, was examined using western blotting (Fig. 3A and B). SH-SY5Y cells differentiated with IGF-I displayed no significant change in the relative expression of ChAT compared with vehicle-treated cells (Fig. 3A). In this experiment, we were unable to detect TH due to insufficient protein expression in undifferentiated and differentiated cells with IGF-I; therefore, TH expression was excluded from the further evaluation (data not shown). In contrast, cells treated with ATRA for 7 days showed a significant increase in the expression of TH, but not ChAT, compared with vehicle-treated cells (Fig. 3B).

### Cell Cycle Changes in SH-SY5Y Cells Differentiated with IGF-I or ATRA

**Fig. 4:**
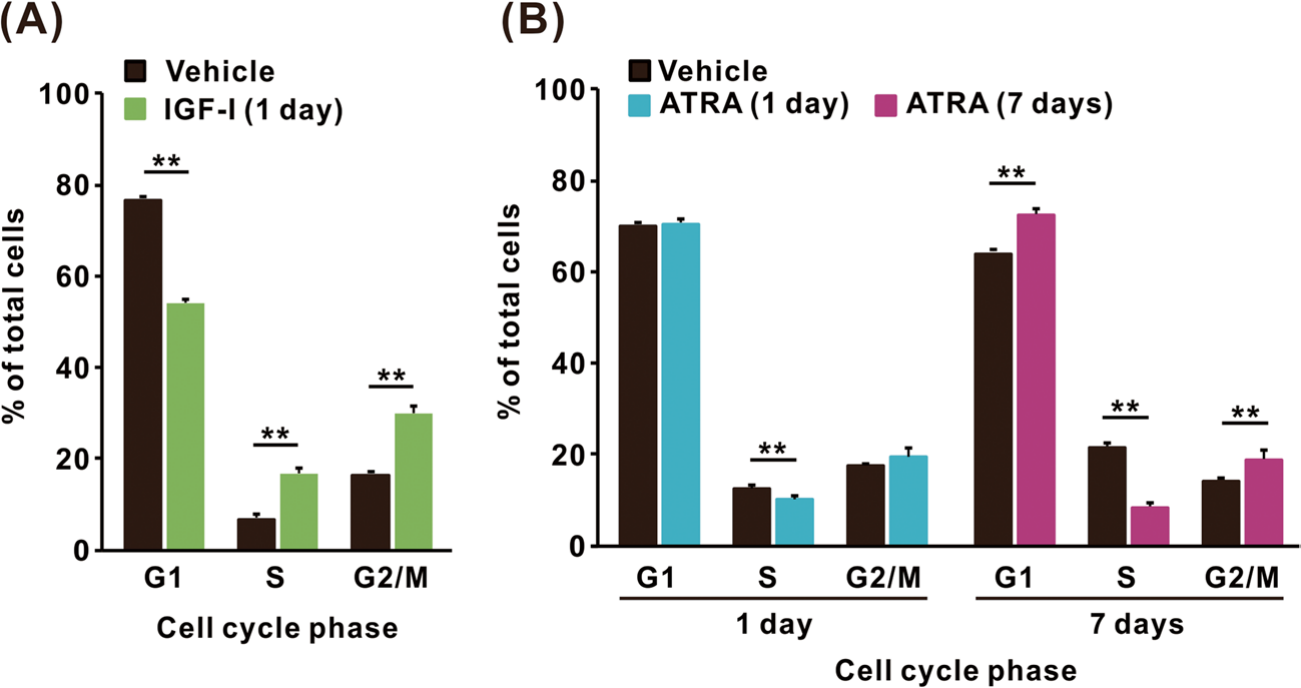
Alterations in the cell cycle in SH-SY5Y cells treated with IGF-I or ATRA. Alteration in the cell cycle was evaluated using flow cytometry in SH-SY5Y cells treated with **A** IGF-I vehicle or IGF-I (50 ng/mL) for 1 day and **B** ATRA vehicle or ATRA (10 µM) for 1 and 7 days. IGF-I was treated in DMEM/F-12 without hiFBS for 1 day, whereas ATRA was treated in DMEM/F-12 containing 1% hiFBS for up to 7 days. Data are expressed as a percentage of the total cells in the G1, S, and G2/M populations. Results are shown as mean ± SEM (*n* = 5). **p < 0.01 compared with vehicle-treated cells

Cell cycle patterns can be altered by differentiation in many cell lines, including SH-SY5Y [42]. Therefore, changes in the subpopulations of SH-SY5Y cells in the G1, S, and G2/M phases were measured after treatment with IGF-I for 1 day or with ATRA for 1 and 7 days (Fig. 4). The proportion of cells in the G1 phase decreased significantly after treatment with IGF-I compared with that in the G1 phase after vehicle treatment (Fig. 4A), and corresponding increases in the S and G2/M phases were detected. In contrast, the proportion of cells in the S phase decreased significantly after treatment with ATRA for 1 day compared with vehicle-treated cells. A decrease in the number of cells in the S phase was also observed in cells treated with ATRA for 7 days, accompanied by a significant increase in the number of cells in the G1 and G2/M phases (Fig. 4B).

### Cytotoxic Effects of Heavy Metals in SH-SY5Y Cells Differentiated with IGF-I or ATRA

**Fig. 5:**
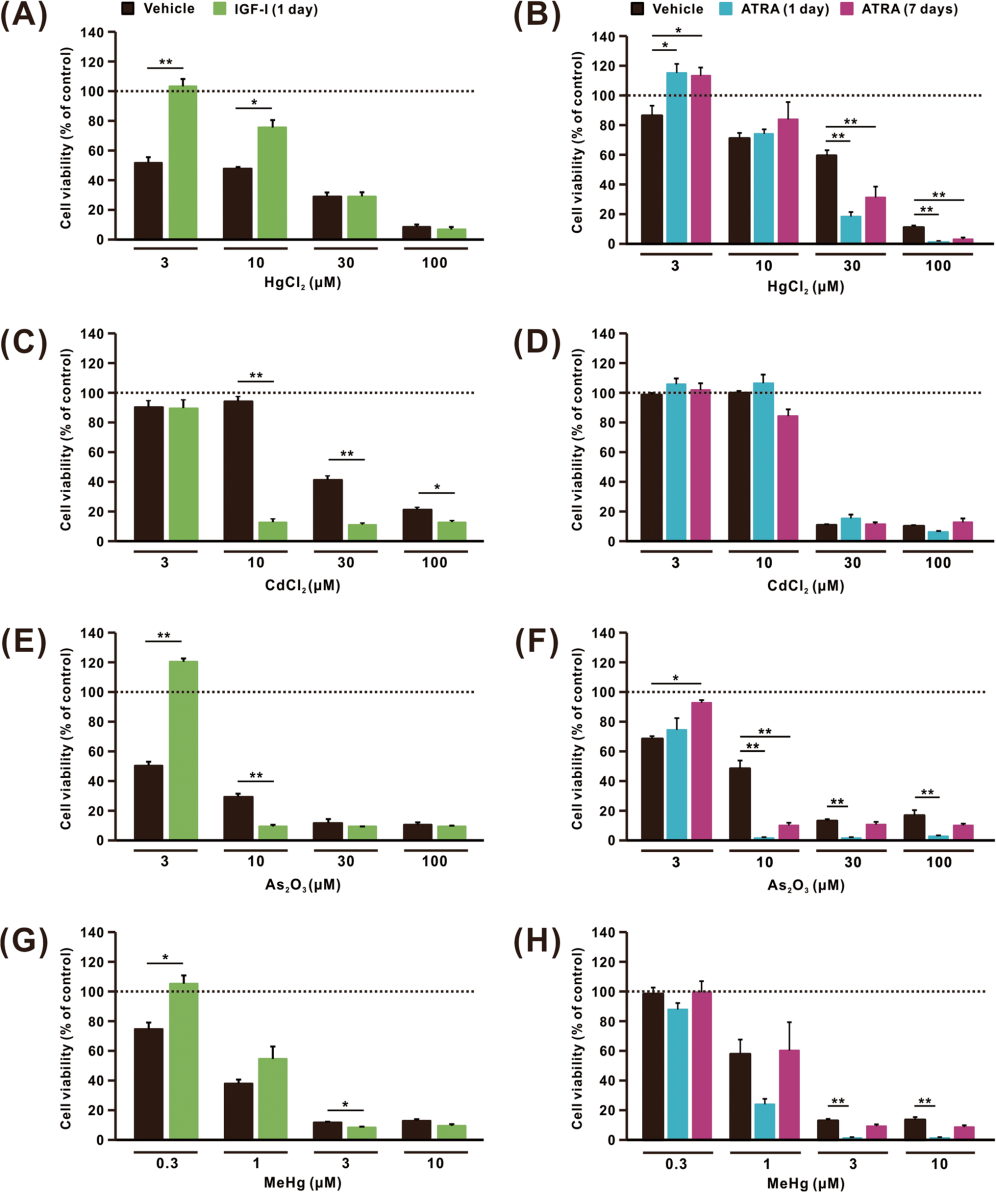
Changes in the viability of differentiated SH-SY5Y cells induced by heavy metals. Cell viability was evaluated at 48 h after exposure to 3–100 µM HgCl_2_ (**A**, **B**), CdCl_2_ (**C**, **D**), As_2_O_3_ (**E**, **F**), and 0.3–10 µM MeHg (**G**, **H**) in cells differentiated with IGF-I (**A**, **C**, **E**, **G**), and ATRA (**B**, **D**, **F**, **H**). Cells without heavy metal treatment were considered to have 100% viability. Results are shown as mean ± SEM (*n* = 5). After conducting two-way ANOVA, the Bonferroni test (**A**, **C**, **E**, **G**) or Dunnett’s test (**B**, **D**, **F**, **H**) was performed. *p < 0.05; **p < 0.01 compared with vehicle-treated cells

The cytotoxic effects of heavy metals on SH-SY5Y cells differentiated with IGF-I or ATRA were compared using cell viability assays (Fig. 5). Undifferentiated (treated with vehicles of IGF-I or ATRA) and differentiated (treated with IGF-I or ATRA) SH-SY5Y cells were exposed to HgCl_2_, CdCl_2_, and As_2_O_3_ (3–100 µM) or MeHg (0.3–10 µM) for 48 h. The decrease in cell viability of IGF-I differentiated cells induced by treatment with 3 and 10 µM HgCl_2_ was significantly lower than that in undifferentiated cells, whereas no difference in sensitivity to 30 and 100 µM HgCl_2_ was observed between undifferentiated and IGF-I-differentiated cell (Fig. 5A). Although the effect of 3 µM HgCl_2_ on cell viability in ATRA-differentiated cells was lower than that in undifferentiated cells, the decrease in cell viability induced by 30 and 100 µM HgCl_2_ was significantly higher than that induced in undifferentiated cells (Fig. 5B). When cells were treated with CdCl_2_, the cytotoxic effect of 10, 30, and 100 µM CdCl_2_ was markedly elevated in IGF-I-differentiated cells compared with that in undifferentiated cells (Fig. 5C). Unlike IGF-I-mediated differentiation, differentiation with ATRA for 1 or 7 days did not alter the cytotoxic effects of CdCl_2_ (Fig. 5D). The decrease in cell viability induced by 3 µM As_2_O_3_ was significantly lower in IGF-I-differentiated cells compared with that in undifferentiated cells; however, increased cytotoxic effects were induced by 10 µM As_2_O_3_ in IGF-I-differentiated cells (Fig. 5E). In ATRA-differentiated cell, increased cytotoxic effects of 10–100 µM As_2_O_3_ were observed in addition to decreased cytotoxic effect of 3 µM. The increased cytotoxic effects of As_2_O_3_ were more evident after 1 day of treatment with ATRA. Finally, a decrease in cell viability was observed after MeHg treatment. In IGF-differentiated cells, the cytotoxic effects of MeHg were lower at 0.3 µM than in undifferentiated cells (Fig. 5G). However, no change was observed in the cytotoxic effects of 0.3 µM MeHg in ATRA-treated cells compared with undifferentiated cells (Fig. 5H). Like As_2_O_3_ treatment, the increased cytotoxic effects of 3 and 10 µM MeHg were more evident in cells differentiated with ATRA for 1 than for 7 days.

## Discussion

In this study, SH-SY5Y cells were differentiated with IGF-I or ATRA to compare the effects of differentiation on the cytotoxicity induced by heavy metals such as HgCl_2_, CdCl_2_, As_2_O_3_, and MeHg. To the best of our knowledge, this is the first study to compare the responses of differentiated SH-SY5Y cells to neurotoxic heavy metals using two distinct differentiation protocols. Neurite outgrowth, neuronal and phenotypic marker expression, and the cell cycle were differentially regulated in cells differentiated with IGF-I or ATRA, suggesting that these differentiation protocols induced differentiated SH-SY5Y cells with distinct characteristics. Interestingly, in differentiated SH-SY5Y cells, diverse changes in sensitivity to heavy metals were observed, depending not only on the differentiation agents and treatment time but also on the heavy metal species and concentration.

Neurite outgrowth, a morphological marker of neuronal differentiation, was detected in SH-SY5Y cells 1 day after treatment with IGF-I and 7 days after treatment with ATRA, as previously reported [19, 21]. We showed that the average neurite length in SH-SY5Y cells differentiated with IGF-I for 1 day was comparable to that in cells differentiated with ATRA for 7 days, suggesting that IGF-I can induce morphological differentiation of SH-SY5Y cells in a shorter period than ATRA. In accordance with neurite elongation, we observed changes in MAP2 and SYP protein levels in SH-SY5Y cells differentiated with IGF-I or ATRA, respectively. The expression of neuronal marker proteins, including MAP2 and SYP, indicates the transformation of immature neuronal cells to mature neuron-like cells through differentiation [43, 44]. Therefore, considering the temporal pattern of neurite outgrowth and expression of a neuronal marker, IGF-I and ATRA induced distinct types of neuronal differentiation in SH-SY5Y cells under our culture conditions. As we showed that the toxicity of heavy metals depended on the type of differentiated cells, the changes of sensitivity to toxicants, including amyloid-β peptide, DSP-4, 5-FU, cisplatin, 6-OHDA, and colchicine, in differentiated SH-SY5Y cells by using various protocols had been reported previously [18, 45, 46]. This implies that, especially for neurons, it is necessary to employ in vitro cell models with appropriate differentiation stages and characteristics to achieve accurate experimental results.

SH-SY5Y cells differentiate into dopaminergic or cholinergic phenotypes depending on the culture conditions, including the agents used to differentiate the cells, [20] and are used as in vitro models for Parkinson’s disease and Alzheimer’s disease research, respectively [47, 48]. Therefore, the expression of TH, a dopaminergic neuronal marker, and ChAT, a cholinergic neuronal marker, was observed using western blotting to evaluate the neuronal phenotypes induced by IGF-I or ATRA. We observed a significant increase in TH expression in SH-SY5Y cells differentiated with ATRA compared with that in undifferentiated cells, in accordance with previous studies [19, 49]. These observations imply that ATRA-differentiated cells show dopaminergic phenotypes, which may account for the differences in heavy metal toxicity in cells differentiated with IGF-I or ATRA. Furthermore, although ATRA-differentiated SH-SY5Y cells showed increased sensitivity to higher concentrations of HgCl_2_, As_2_O_3_, and MeHg compared with undifferentiated cells, increased sensitivity to these heavy metals was more evident in cells differentiated with ATRA for 1 than for 7 days. These results suggest that the sensitivity to heavy metals varies with the phenotypic changes induced by ATRA and the degree of differentiation. Heavy metals, including mercury, cadmium, and arsenic, have been reported to be significant pathogenic factors in Parkinson’s disease, a disorder arising from the degeneration of dopaminergic neurons [50, 51]. More detailed studies on the relationship between neuronal phenotypes and heavy metal toxicity using an appropriate differentiated neuronal cell model are needed to better understand the pathologies associated with heavy metal exposure.

Since alterations in the cell cycle associated with cell differentiation have been reported [25, 26, 52], the differences in cell cycle alterations in SH-SY5Y cells differentiated with IGF-I or ATRA were determined. Differentiation of cells with IGF-I induced a significant decrease in the proportion of cells in the G1 phase and an increase in cells in the S and G2/M phases. ATRA induced a slight decrease in the proportion of cells in the S phase after 1 day of treatment. An accumulation of cells in the G1 and G2/M phases and a gradual reduction in the S phase were observed with the progression of ATRA-mediated differentiation. These results suggest that the cell cycle is differentially regulated during neuronal differentiation induced by IGF-I or ATRA and support the idea that IGF-I and ATRA induce distinct types of neuronal differentiation in SH-SY5Y cells. In the cell viability study, we observed that the cadmium sensitivity of IGF-differentiated SH-SY5Y cells was significantly higher than that of undifferentiated cells. Recently, the effects of cadmium on NRK-52E cells, a rat renal proximal tubular cell line, at various growth phases have been reported; G0 and S-phase cells were more prone to apoptosis, S- and M-phase cells were more prone to necrosis, and G1 cells were less affected. Because the induction of apoptosis and necrosis by cadmium has been reported in neuronal cells [53–55], the cell cycle changes induced by IGF-I treatment may explain the increased sensitivity to cadmium in IGF-I-induced differentiated cells through the modification of the cytotoxic effects of apoptosis and necrosis.

A reduction in heavy metal cytotoxicity was observed at low concentrations in SH-SY5Y cells differentiated with IGF-I or ATRA. A possible reason for this phenomenon is that these heavy metals may exert toxic effects on the proliferation of undifferentiated SH-SY5Y cells, which have a higher proliferation rate than differentiated cells [18]. In addition, IGF-I is known to be neuroprotective against many toxic effects [35–39]. Since IGF-I exerts neuroprotective effects via the activation of the PI3-kinase pathway, which is also important for the induction of neurite outgrowth in SH-SY5Y cells [56, 57], IGF-I-differentiated cells may develop tolerance to the toxicity of low concentrations of heavy metals.

In this study, we focused on evaluating heavy metal–related cytotoxicity in SH-SY5Y cells after ATRA- and IGF-I-induced differentiation. However, this focus on cytotoxicity represents a limitation of this study, in that our results are not sufficient to explain all mechanisms involved in the diverse changes that heavy metals can induce. Heavy metals reportedly commonly induce oxidative stress through disruption of cellular redox homeostasis, with mercury, cadmium, and arsenic among the elements exerting this toxic effect [58]. Such stress can result in irreversible oxidative modifications in any biomolecule, ranging from lipids to DNA and proteins [59]. And inducing oxidative stress, mercury, cadmium, and arsenic also reportedly affect undifferentiated SH-SY5Y cells, with cell cycle dysregulation, decreased cell proliferation, and caspase-dependent apoptosis playing roles in the underlying mechanisms of cytotoxicity [10, 32, 34, 60–65]. Further investigation of these mechanisms in differentiated SH-SY5Y cells will facilitate a more comprehensive understanding of the impact of differentiation protocols on heavy metal–related cytotoxicity.

## Conclusion

The present study demonstrates that in vitro differentiation protocols influence the response of SH-SY5Y cells to heavy metals. In differentiated SH-SY5Y cells, diverse changes in cytotoxicity to heavy metals compared with undifferentiated cells were detected, depending on the agents and treatment period for differentiation, as well as the heavy metal species and concentrations. Our results provide new insights into the assessment of neurotoxic heavy metals. Differentiated SH-SY5Y cells induced by various routes are useful tools for elucidating the mechanism of toxicity of neurotoxic heavy metals. Elucidating the characteristics of the cytotoxic effects in distinct types of differentiated SH-SY5Y cells may help clarify the precise cytotoxic mechanisms, effects on health, and development of effective therapies for heavy metal poisoning. For future research on the cytotoxicity of heavy metals, signaling pathways should be investigated using an appropriate differentiation model to obtain accurate results comparable to in vivo models.

## Data Availability

The data that support the findings of this study are available from the corresponding author, upon reasonable request.
